# Genome-wide loss of heterozygosity predicts aggressive, treatment-refractory behavior in pituitary neuroendocrine tumors

**DOI:** 10.1007/s00401-024-02736-8

**Published:** 2024-05-17

**Authors:** Andrew L. Lin, Vasilisa A. Rudneva, Allison L. Richards, Yanming Zhang, Hyung Jun Woo, Marc Cohen, Jamie Tisnado, Nazanin Majd, Sharon L. Wardlaw, Gabrielle Page-Wilson, Soma Sengupta, Frances Chow, Bernard Goichot, Byram H. Ozer, Jorg Dietrich, Lisa Nachtigall, Arati Desai, Tina Alano, Shahiba Ogilive, David B. Solit, Tejus A. Bale, Marc Rosenblum, Mark T. A. Donoghue, Eliza B. Geer, Viviane Tabar

**Affiliations:** 1 Department of Neurosurgery, Memorial Sloan Kettering Cancer Center, 1275 York Avenue, New York, NY 10065 USA; 2 Department of Neurology, Memorial Sloan Kettering Cancer Center, New York, NY USA; 3 Multidisciplinary Pituitary and Skull Base Tumor Center, Memorial Sloan Kettering Cancer Center, New York, NY USA; 4 Marie-Josée and Henry R. Kravis Center for Molecular Oncology, Memorial Sloan Kettering Cancer Center, New York, NY USA; 5 Department of Pathology and Laboratory Medicine, Memorial Sloan Kettering Cancer Center, New York, NY USA; 6 Department of Surgery, Memorial Sloan Kettering Cancer Center, New York, NY USA; 7 Department of Radiology, Memorial Sloan Kettering Cancer Center, New York, NY USA; 8 Department of Neuro-Oncology, The University of Texas MD Anderson Cancer Center, Houston, TX USA; 9 Department of Medicine, Columbia University Irving Medical Center, New York, NY USA; 10 Department of Neurology and Neurosurgery, University of North Carolina, Chapel Hill, NC USA; 11 Department of Neurology, Keck School of Medicine at University of Southern California Medical Center, Los Angeles, CA USA; 12 Department of Endocrinology, Les Hôpitaux Universitaires de Strasbourg, Strasbourg, France; 13 Department of Oncology, Sibley Memorial Hospital/Johns Hopkins, Washington, DC USA; 14 Department of Neurology, Massachusetts General Hospital, Boston, MA USA; 15 Department of Medicine, Massachusetts General Hospital, Boston, MA USA; 16 Department of Medicine, University of Pennsylvania Medical Center, Philadelphia, PA USA; 17 Department of Medicine, Memorial Sloan Kettering Cancer Center, New York, NY USA

**Keywords:** Aggressive pituitary tumor, Treatment-refractory pituitary tumor, Pituitary carcinoma, Pituitary neuroendocrine tumor

## Abstract

Pituitary neuroendocrine tumors (PitNETs) exhibiting aggressive, treatment-refractory behavior are the rare subset that progress after surgery, conventional medical therapies, and an initial course of radiation and are characterized by unrelenting growth and/or metastatic dissemination. Two groups of patients with PitNETs were sequenced: a prospective group of patients (*n* = 66) who consented to sequencing prior to surgery and a retrospective group (*n* = 26) comprised of aggressive/higher risk PitNETs. A higher mutational burden and fraction of loss of heterozygosity (LOH) was found in the aggressive, treatment-refractory PitNETs compared to the benign tumors (*p* = 1.3 × 10^−10^ and *p* = 8.5 × 10^−9^, respectively). Within the corticotroph lineage, a characteristic pattern of recurrent chromosomal LOH in 12 specific chromosomes was associated with treatment-refractoriness (occurring in 11 of 14 treatment-refractory versus 1 of 14 benign corticotroph PitNETs, *p* = 1.7 × 10^−4^). Across the cohort, a higher fraction of LOH was identified in tumors with *TP53* mutations (*p* = 3.3 × 10^−8^). A machine learning approach identified loss of heterozygosity as the most predictive variable for aggressive, treatment-refractory behavior, outperforming the most common gene-level alteration, *TP53*, with an accuracy of 0.88 (95% CI: 0.70–0.96). Aggressive, treatment-refractory PitNETs are characterized by significant aneuploidy due to widespread chromosomal LOH, most prominently in the corticotroph tumors. This LOH predicts treatment-refractoriness with high accuracy and represents a novel biomarker for this poorly defined PitNET category.

**Supplementary Information:**

The online version contains supplementary material available at 10.1007/s00401-024-02736-8.

## Introduction

Pituitary neuroendocrine tumors (PitNETs) are largely benign. The small subset that requires intervention are typically addressed with standard treatments; only a minor percentage of clinically significant PitNETs are difficult to control and are considered aggressive.

The European Society of Endocrinology (ESE) Clinical Practice Guidelines define aggressive PitNETs as tumors that are invasive and grow at an unusually rapid rate, or progress in spite of standard treatment (surgery, conventional medical therapies, and radiotherapy) [41]. While a consensus on the definition of treatment-refractory behavior has not been reached [18], aggressiveness as defined by the ESE incorporates a key feature of treatment-refractory behavior (progression despite standard interventions). For this reason, a PitNET that progresses on imaging following surgery, conventional medical therapies, and radiotherapy should be considered both aggressive and treatment-refractory.

Aggressive, treatment-refractory behavior is rare (occurring in  < 1% of PitNETs). These tumors can be locally destructive, cause significant morbidity and mortality, and can become metastatic. At present, there are no known histopathologic or molecular markers that robustly predict future treatment-refractory behavior. The 2017 WHO classification abandoned the atypical designation, which was based on TP53 overexpression by immunohistochemistry (IHC), the presence of an elevated mitotic index, and a Ki-67 labeling index greater than 3% [1]. This designation was eliminated after it was shown that this grading schema did not predict aggressive behavior [37].

To predict the tumors at highest risk of recurrence, Trouillas et al. devised a clinicopathological scoring system that designates a “grade” based on evidence of invasion by histology/imaging and markers of proliferation (Ki-67, mitotic index, and TP53 overexpression) [49]. In their analysis, grade was associated with patient progression/recurrence status after 8 years. Independent data sets have confirmed that this classification system predicts progression-free survival, but it has not been shown to identify the subset of tumors that will progress following maximal therapy including radiation [4, 42]. While a few genetic biomarkers of aggressive behavior have been proposed, the data are conflicting and limited by insufficient follow-up. Higher frequency of loss of heterozygosity at microsatellite markers has been associated with recurrence and invasiveness [6, 11], and higher degrees of copy-number alterations have been associated with worse outcome in some but not all studies [26, 38, 46, 50]. Additionally, an enrichment of *TP53* mutations has been observed in subtypes of corticotroph tumors with higher risk clinicopathological features [50], *SF3B1* R625H mutations have been identified in rapidly progressive lactotroph PitNETs [28], and mutations in *ATRX* and *DAXX* have been reported in recurrent PitNETs [12, 20]. However, it remains unknown if these alterations are associated with a treatment-refractory phenotype.

Given that most patients with PitNETs are controlled by the conventional treatments, a validated biomarker of poor prognosis is required to study more intensive treatments at an earlier timepoint. Moreover, a prognostic biomarker could improve outcomes by permitting de-escalation of treatment for patients with tumors that are currently considered high risk based on clinicopathologic features yet follow a benign clinical course. To identify molecular signatures that characterize tumors with a worse prognosis, we identified a set of patients with proven aggressive, treatment-refractory PitNETs or higher risk features and performed comprehensive genomic analyses on their tumors and compared them to a prospectively collected cohort of patients with PitNETs who presented to Memorial Sloan Kettering Cancer Center (MSKCC) for a transsphenoidal resection.

## Materials and methods

Two groups of patients were enrolled: (1) a prospective, unselected collection of patients (*n* = 66) who presented to MSKCC for pituitary surgery and provided written informed consent to the sequencing study prior to surgery, and (2) a retrospective collection of pituitary patients (*n* = 26) whose tumors had either demonstrated aggressive, treatment-refractory behavior as defined by progression on MRI following standard treatments including a first course of external beam radiation (22/26, 85%) or were considered at a higher risk as per the 2017 and/or 2022 World Health Organization classification of pituitary tumors [52]: a silent corticotroph PitNET, lactotroph PitNET in a man, Crooke’s cell PitNET, or immunonegative PitNET (4/26, 15%). Corticotroph tumors were considered biochemically silent by fulfilling one of the following criteria: (1) normal 24 h urine free cortisol, (2) diagnosis of adrenal insufficiency requiring glucocorticoid replacement, or (3) confirmation by referring endocrinologist. For the patients in the prospective cohort, the pre- and post-surgical imaging was reviewed, and the Knosp score, longest diameter, and the extent of resection were determined. For both cohorts, the treatment history including the number of surgeries, courses of radiotherapy, and lines of medical therapy was collected. Tumor tissue from all patients enrolled on the sequencing protocol was analyzed by an experienced neuropathologist (either MR or TB).

### Immunohistochemistry

Formalin-fixed paraffin-embedded tissue underwent hematoxylin and eosin staining as well as immunohistochemistry to establish the tumor’s histopathological classification and mismatch repair status. Rabbit polyclonal primary antibodies (Roche Ventana, Tucson, AZ) were used in the detection of ACTH, LH, FSH, TSH, prolactin, growth hormone, P53, and Ki-67 on a Benchmark Ultra (Roche Ventana, Tucson AZ). Monoclonal antibodies from clone N1665 (Thermo Fisher Scientific, Rockford, IL) were used to detect SF-1; monoclonal antibodies from clone CL6251 (Sigma-Aldrich, St. Louis, MO) were used to detect T-PIT/TBX19; monoclonal antibodies from clone 8B6.1 (EMD Millipore, Burlington, MA) were used to detect PIT-1; monoclonal antibodies from clone ES05 (Leica Biosystems, Wetzlar, Germany) were used to detect MLH1; monoclonal antibodies from clone G21-91129 (Cell Marque, Rocklin, CA) were used to detect MSH2; monoclonal antibodies from clone EP49 (Agilent Dako, Santa Clara, CA) were used to detect MSH6; and monoclonal antibodies from clone A16-4 (BD Bioscience, San Diego, CA) were used to detect PMS2 on a Bond-III IHC and ISH stainer (Leica Biosystems, Wetzlar, Germany). In the treatment-refractory cohort, Ki-67 from the diagnostic resection was extracted from pathology reports when banked tissue was unavailable due to the age of these samples.

### Genomic sequencing and analysis

All tumors in the prospective and retrospective cohorts underwent next-generation sequencing via MSK-IMPACT targeted sequencing panel, with somatic mutations (substitutions, small insertions, and deletions), gene-level focal CNAs, and selected structural rearrangements detected with a clinically validated pipeline as previously described [13, 53]. In brief, tumor DNA was extracted from banked formalin-fixed paraffin-embedded tumor specimens and normal DNA was extracted from mononuclear cells in patient-matched peripheral blood, saliva, or finger or toenail samples. Using MSK-IMPACT, we evaluated for recurrent somatic alterations classified as oncogenic or likely oncogenic using OncoKB [53]. Tumor mutational burden (TMB) was defined as the number of non-synonymous mutations in canonical exons per megabase. Prior sequencing efforts involving PitNETs described gain-of-function mutations in *USP8* as a recurrent event in PitNETs. Since *USP8* gene is not present on MSK-IMPACT panels used in this study, we used whole-exome sequencing recapture data to call these mutations in the retrospective cohort of patients as previously described [23]. Specifically, the MSK-IMPACT cDNA libraries underwent target capture using SureSelect Human All Exon V6 (Agilent Technologies, Santa Clara, CA) and then underwent whole-exome sequencing on a HiSeq 2500 (Illumina, San Diego, CA). Whole-exome sequencing data were processed and analyzed using the TEMPO pipeline (v1.3, https://github.com/mskcc/tempo). In brief, demultiplexed FASTQ files were aligned to the b37 assembly of the human reference genome from the GATK bundle using BWA mem (v0.7.17) [29]. Aligned reads were converted and sorted into BAM files using samtools (v1.9) [14] and marked for PCR duplicates using GATK MarkDuplicates (v3.8-1) [16, 36, 51]. Somatic mutations (single-nucleotide variants and small insertions and deletions) were called in tumor-normal pairs using MuTect2 (v4.1.0.0) [8] and Strelka2 (v2.9.10) [24]. Variants were annotated and filtered for recurrent artifacts and false positives using methods as previously described [23]. Mutational signatures were determined via maximum-likelihood-based extraction of mutational signature proportions of a set of mutation count data under a known set of inputs signature lists (“refitting”, https://github.com/mskcc/tempoSig). The microsatellite instability status was determined using MiMSI, a classifier that is better suited for assessing microsatellite instability status in tumors with a high fraction of genome altered (https://github.com/mskcc/mimsi) [55]. MiMSI-Status is determined based on the lower CI and upper CI intervals with the following criteria: LCI and UCI < 0.5 = MSS, LCI and UCI > 0.5 = MSI-H, and LCI < 0.5 UCI =  ~ 0.5 (meaning that there is no congruency between the two values) = MSI-I (indeterminate).

Allele-specific copy-number analysis was performed using FACETS version 0.5.6 [45]. FACETS fits were manually reviewed. Fraction of genome altered, fraction of loss of heterozygosity (LOH), and LOH status of individual chromosomes were inferred using “facets-suite” utility from MSKCC (https://github.com/mskcc/facets-suite). Tumors were considered fraction of genome-high (FGA-H) and fraction of LOH-high (LOH-H) if they are above the corresponding median (0.22 for FGA and 0.03 for LOH) for the cohort. The expected number of copies for each mutation was generated based on observed variant allele fraction and local ploidy [15]. Cancer cell fractions (CCF) were calculated using a binomial distribution and maximum-likelihood estimation normalized to produce posterior probabilities [35]. Copy-number events (amplifications and deletions) and fusions were manually reviewed. Mutations were considered clonal if the CCF > 0.8 or CCF > 0.7 and the CFF upper C.I. > 0.9. The largest chromosomal segment with LOH was used to estimate the cellular fraction of a chromosomal loss. To adjust for errors in the estimation of the CF, all chromosomal CF values above 90% of the maximal per sample CF value were considered the same, and thus the corresponding chromosomal losses as occurring in the same proportion of cells.

### Formalin-fixed paraffin-embedded fluorescence in situ hybridization experiments

We performed fluorescence in situ hybridization (FISH), using probes on relevant chromosomal segments, to confirm that loss of heterozygosity is due to the loss of a chromosome arm. Formalin-fixed paraffin-embedded (FFPE) tissue sections of 4 µm thickness with tumor areas marked were used for FISH analysis following standard protocols. Four or more FISH probes were selected for each case to confirm the copy numbers of those chromosomes with LOH features based on MSK-IMPACT FACETS calling [45], including probes for 1p/1q, MYCN/CEP2, MET/CEP7, CDKN2A/CEP9, MDM2/CEP12, NTRK3, FUS, 19p/19q, and EWSR1 on chromosomes 1, 2, 7, 9, 12, 15, 16, 19, and 22 (from Abbott Molecular, Des Plaines, IL; Agilent, Santa Clara, CA; Cytotest, Rockville, MD). Signal analysis was performed in combination with morphology correlation, and at least 50 interphase cells within the marked tumor area were evaluated. Representative FISH images were captured using a Zeiss fluorescence microscope coupled with Metasystems ISIS software (Newton, MA).

### Machine learning

A random forest-based classifier was built using the randomForest R package, which is based on the algorithm of Breiman and Cutler [10]. Random forest is a machine learning method that constructs decision trees for every sample from the provided dataset. Each decision tree provides a prediction, and a final prediction is made based upon majority vote. The random forest model was trained as follows: the dataset was randomly split into a training and test set at a ratio of 7:3. We first selected genomic and clinical features to be used in our RF model: fraction CNA, fraction LOH, gender, lineage, TP53 status of the earliest sample, and LOH status for chromosomes 1 through 22. In total, these 27 features were evaluated in the training dataset for inclusion in the final classifier algorithms using nearZeroVar function from the caret package that identifies predictors with near zero variance. LOH status for chromosomes 5, 7, 12, 14, and 20 were diagnosed as near zero variance predictors and therefore excluded from the model. The model was trained on the training set, with 500 trees and 22 variables to select randomly for each tree. Validation of the classifier was performed on the training set, and out-of-bag (OOB) error was used to measure the model’s error rate. Random forest classifier performance was evaluated using receiver-operating characteristic under the curve (ROC-AUC) and accuracy based on the test set. Random forests can be used to rank the importance of variables. For variable selection, features were ranked based on mean minimal depth, number of times this variable is a root, and the number of nodes. Cutpointr R package was used to choose an optimal threshold for fraction of LOH value based on maximizing the sum of sensitivity and specificity [48].

### Statistics

Statistical tests were performed using R, version 4.3.1. Statistical analyses was performed using a one- or two-sided Wilcoxon rank sum or Fisher’s exact test as reported. *P* values of less than 0.05 indicates statistical significance.

## Results

### Demographics

**Fig. 1:**
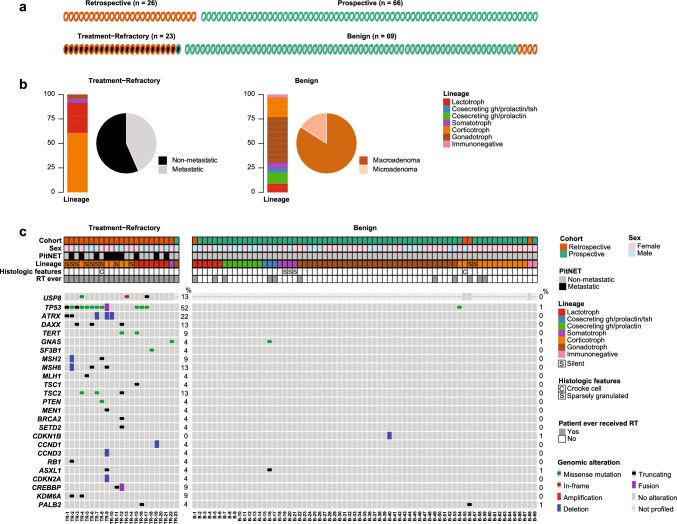
Genomic alterations and clinical characteristics of patients with aggressive, treatment-refractory and benign PitNETs*.* (**a**) Schematic diagram showing relationship between the retrospective and prospective groups and clinical behavior. (**b**) Summary of the tumor lineages in the treatment-refractory and benign subsets and metastatic disease status at the time of the data freeze (treatment-refractory subset). (**c**) Oncoprint summarizing recurrently altered driver genes in treatment-refractory (left: *n* = 23) and benign (right: *n* = 69) PitNETs. Patients who had multiple tumor samples are represented by the union of alterations among all samples. Patient demographics and clinicopathologic features are on the top, followed by common genetic alterations (frequencies are shown on the right in percent per clinical category). *USP8* status is reported if whole-exome recapture was performed

The retrospective group is comprised of 26 patients and includes patients whose tumors were either treatment-refractory or considered higher risk based on clinicopathologic features, Fig. 1a and Table 1. Twenty-four (92%) of these patients received radiation and 20 (77%) received treatment with the alkylator chemotherapeutic agent, temozolomide, during their clinical course; the median number of surgeries was three (range = 1–10). Of these 26 patients, 22 (85%) had PitNETs that were treatment-refractory as characterized by progression following conventional treatments, including a first course of radiotherapy. Among these aggressive, treatment-refractory tumors, ten (45%) were metastatic, Fig. 1b. Eight of the ten patients with metastatic PitNETs had systemic metastases, of which five were corticotroph and three were lactotroph PitNETs; two of the ten had leptomeningeal dissemination and were corticotroph tumors. Fourteen patients with treatment-refractory PitNETs had corticotroph (59%), seven had lactotroph (32%), and one had a somatotroph tumor, Fig. 1b. Among the treatment-refractory corticotroph tumors, nine patients (64%) had clinically silent tumors without evidence of hypercortisolemia at the time of surgery. Among patients with treatment-refractory lactotroph PitNETs, four (57%) were male, Fig. 1c. For 19 of 23 patients, the Ki-67 at the time of diagnosis is available; the Ki-67 was 3% or less in 6, 10% or less in 5, and  > 10% in 7 (the final patient was reported to have a low proliferative index without quantification).

**Table 1:** Characteristics of the retrospective cohort

Characteristic	Number of patients (*n* = 26)	Percentage
*Median age at diagnosis*	41	
*Metastatic*		
No	16	62%
Yes	10	38%
*Gender*		
Male	12	46%
Female	14	54%
*Histology*		
Corticotroph	16	62%
Lactotroph	8	31%
Immunonegative	1	4%
Somatotroph	1	4%
*Treatment-refractory*		
Yes	22	85%
No	4	15%
*Surgeries*		
1 surgery	3	12%
2 surgeries	9	35%
3 surgeries	4	15%
4 + surgeries	10	38%
*Radiation at any time*		
Yes	24	92%
No	2	8%
*Received temozolomide*		
Yes	20	77%
No	6	23%

The prospective group consists of 66 patients who presented to MSKCC for resection of their PitNET and were consented to the sequencing protocol prior to surgery, Fig. 1a and Table 2. The characteristics of this cohort are typical of the patient population that seeks a surgical opinion at a quaternary care hospital and based on these characteristics, it is comprised of tumors with low malignant potential. In these patients, the median number of surgeries was one (range = 1–5). Of these 66 patients, only one patient was treatment-refractory as defined by progression on imaging following standard treatments, including an initial course of radiotherapy, Fig. 1a. Notably, this patient with an enlarging gonadotroph PitNET after radiotherapy had a cystic tumor, which was found to be largely comprised of blood products in the operating room. Retrieval of these blood products permitted a complete radiographic resection, and this patient has been disease free for 5 years. None of these surgical patients had metastatic disease. The majority of resected tumors were macroadenomas (83%; *n* = 55), Fig. 1b. During their clinical course, ten (15%) of the cohort received radiation either as adjuvant treatment or due to tumor progression, and only one patient received temozolomide. This patient received temozolomide instead of radiotherapy because optic nerve atrophy placed her at higher risk for radiation-induced optic neuropathy.

**Table 2:** Characteristics of the prospective cohort

Characteristic	Number of patients (*n* = 66)	Percentage
*Median age at diagnosis*	51	
*Metastatic*		
No	66	100%
Yes	0	0%
*Gender*		
Male	27	41%
Female	39	59%
*Histology*		
Corticotroph	12	18%
Co-secreting GH/prolactin	8	12%
Co-secreting GH/prolactin/TSH	3	5%
Lactotroph	5	8%
Gonadotroph	33	50%
Immunonegative	1	2%
Somatotroph	4	6%
*Treatment-refractory*		
Yes	1	2%
No	65	98%
*Surgeries*		
1 surgery	53	80%
2 surgeries	9	14%
3 + surgeries	4	6%
*Radiation at any time*		
Yes	10	15%
No	56	85%
*Received temozolomide*		
Yes	1	2%
No	65	98%
*Size of profiled tumor*		
Macroadenoma	55	83%
Microadenoma	11	17%
*Knosp score of profiled tumor*		
0	28	42%
1	17	26%
2	9	14%
3A	5	8%
4	7	11%
*Ki67 of profiled tumor*		
< 3%	58	88%
> 3% and 10% or less	6	9%
Not available	2	3%
* Extent of resection of profiled tumor*		
Subtotal resection	15	23%
Gross total resection	46	70%
Near gross total resection	5	8%

Among the 23 patients with aggressive, treatment-refractory PitNETs (retrospective cohort: *n* = 22, prospective cohort: *n* = 1), the median time from diagnosis to radiation was 0.61 years with a range of 0.25–13.3 years, as some patients received adjuvant radiation, while others were irradiated after demonstrating progression of disease. The median time from radiation to first progression was 2.8 years with a range of 0.5 to 17 years. Metastases developed in ten of the 23 patients with a treatment-refractory tumor at a median of 7.3 years following diagnosis (range of 0.8 to 22.3 years). Among the ten metastatic PitNETs, the median survival from the identification of metastatic disease was 75 months. Of the six patients who died from their treatment-refractory PitNET, one patient died from a tumor that never metastasized but was locally invasive into the brain; the remaining five patients died of metastatic tumors. Salvage treatments after temozolomide included checkpoint inhibitors, lutetium-177 DOTATATE, cisplatin/etoposide, carboplatin/etoposide, everolimus, and lapatinib, and have been described in case reports [19, 30–34, 44].

### Landscape of recurrent somatic alterations at the gene level

Patients from both the retrospective and prospective groups were stratified into two subsets based on their clinical response to radiotherapy: treatment-refractory tumors that progressed following an initial course of radiotherapy (22 from the retrospective and 1 patient from the prospective group) and benign tumors (65 from the prospective and 4 patients from the retrospective group), Fig. 1a, c. Ten of the 23 treatment-refractory patients had at least one sample collected prior to treatment with radiation. Eight treatment-refractory patients had samples from more than one timepoint; shared oncogenic mutations could be identified in five of these patients, Supplementary Fig. 1a. Compared to the benign subset, the aggressive, treatment-refractory PitNETs had more oncogenic or likely oncogenic somatic alterations (tumor mutational burden was higher in the treatment-refractory tumors by a one-sided Wilcoxon rank sum, *p* = 1.3 × 10^−10^), Fig. 1c. The most common alterations were mutations in *TP53*, which were overwhelmingly found in the treatment-refractory tumors (12 of 23 treatment-refractory tumors *vs* 1 of 69 benign tumors, two-sided Fisher’s exact test: *p* value = 4.2 × 10^−8^). Of the 12 PitNETs with *TP53* mutations, 11 were clonal based on cancer cell fraction calculations (see methods). In four of eight corticotroph and one of three lactotroph PitNETs with a *TP53* mutation, we sequenced the tissue specimen from the first resection and the *TP53* mutation was present. These findings are congruent with prior literature, which suggests that *TP53* mutations are most commonly early events but can also develop later in the natural history by convergent evolution [39, 40, 50].

Five aggressive, treatment-refractory corticotroph tumors contained an oncogenic or likely oncogenic alteration in mismatch repair genes (*MSH2*, *MSH6*, and *MLH1,* Fig. 1c). Four of the five PitNETs had alterations in the treatment naïve diagnostic resection; the remaining patient was found to have an MMR alteration in a recurrent tumor that was exposed to temozolomide, which was not present in the pre-treatment resection specimens (TR-9, Supplementary Fig. 1a). To evaluate the significance of the MMR alterations, MMR IHC was performed, confirming loss of protein expression in the mutated MMR gene(s) in all five patients. Furthermore, three of the four patients with a mismatch repair alteration in a pre-treatment sample were microsatellite instability-high (MSI-high) by MiMSI [55]; the remaining patient’s tumor was microsatellite instability-indeterminant (MSI-indeterminant) and had an elevated mutation burden (range for these four specimens: 8.78–12.29 mutations/Mb). Only the tumor that developed mismatch repair deficiency as a consequence of therapeutic pressure from temozolomide was microsatellite stable; this tumor was hypermutated with a tumor mutational burden of 93 mutations/Mb. As previously reported, mutational signature decomposition analysis revealed that 76% of the mutations were attributable to temozolomide, which causes a large number of C > T/G > A transitions [2, 32]. Other alterations in the treatment-refractory PitNETs included mutations in genes involved in telomere maintenance (*ATRX*, *DAXX*, and *TERT*) and *TSC2*, Fig. 1c. These alterations were not found in the benign tumors and were unique to the treatment-refractory corticotroph PitNETs.

We next evaluated our cohort for known recurrent mutations, such as *GNAS* R201, which occurs in somatotroph PitNETs [25]. Of the five somatotrophs in our cohort, only the treatment-refractory tumor harbored this alteration. Only one of the seven treatment-refractory lactotrophs, and none of the benign lactotrophs, harbored a hotspot mutation in *SF3B1 R625C*, which was previously reported in prolactinomas with a more aggressive clinical phenotype [28]. Because *USP8* is not included on MSK-IMPACT, we performed whole-exome sequencing on specimens from all patients in the retrospective cohort, which included 22 of the 23 aggressive, treatment-refractory tumors (and all the treatment-refractory corticotroph tumors). *USP8* mutations were rarely identified among the aggressive, treatment-refractory PitNETs (*n* = 3), Fig. 1c and Supplementary Fig. 1b. The solitary tumor with a canonical *USP8* gain-of-function mutation in the 14-3-3 binding domain is from a patient with a treatment-refractory corticotroph PitNET who received two courses of proton RT in 2007 and 2009 with a complete response after the second course, and no recurrence on imaging after over 14 years of radiographic follow-up. The remaining two *USP8* mutations were a missense mutation outside the 14-3-3 binding motif and a truncating mutation in a lactotroph.

### Recurrent genome-wide loss of heterozygosity in treatment-refractory PitNETs

**Fig. 2:**
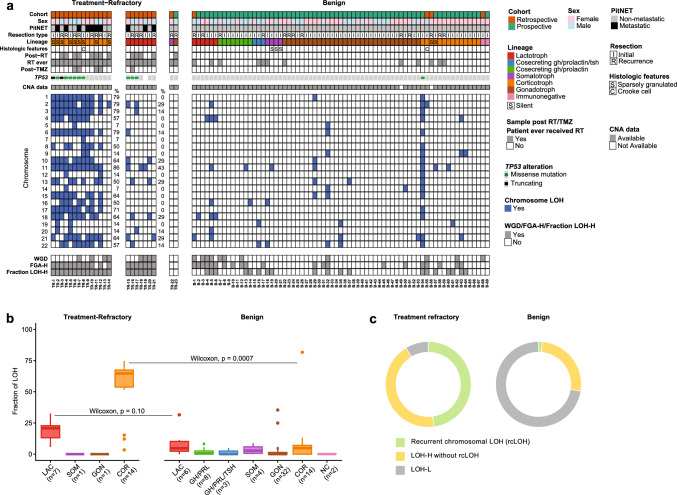
Genomic instability due to loss of heterozygosity (LOH) is frequent in aggressive, treatment-refractory PitNETs. (**a**) The heatmap shows LOH status for individual chromosomes with each blue box demonstrating LOH covering at least 75% of the given chromosome and reflects the first sequenced resection. Top tracks report clinicopathologic features such as lineage and treatment, again for the first sequenced resection. Bottom tracks report features of genomic instability, including whole-genome duplication status (WGD), fraction of genome altered (FGA), and fraction of LOH based on median values (median FGA = 0.22, median fraction of LOH = 0.03). Copy-number alteration (CNA) data are unavailable for two patients as annotated. (**b**) Cohort segregated into treatment-refractory and benign, displaying the fraction of LOH by lineage. Two-sided Wilcoxon rank sum tests are shown. (**c**) The percentage of samples with classifications of LOH: LOH-H (fraction LOH higher than the median = 0.03) with or without rcLOH (involving chromosomes 1, 2, 3, 4, 6, 10, 11, 15, 17, 18, 21, and 22), or LOH-L (fraction of LOH < 0.03)

While the aggressive, treatment-refractory tumors had a higher frequency of gene mutations than the benign tumors, not all treatment-refractory cases were explained by gene-level alterations. Collectively, only 61% (15/23) of patients had oncogenic or likely oncogenic mutations demonstrated previously to be associated with worse prognosis: *TP53*, *ATRX*, *DAXX*, or *SF3B1* [12, 20, 39, 50]. Based on these observations, we hypothesized that alternative molecular features contribute to aggressive, treatment-refractory behavior. To explore this further, we performed allele-specific copy-number analysis using FACETS to infer fraction of genome altered (FGA) and fraction of LOH [45], Fig. 2a. We found a higher FGA and fraction of LOH in the treatment-refractory PitNETs compared to the benign tumors (one-sided Wilcoxon rank sum: *p* = 7.3 × 10^−6^ and *p* = 8.5 × 10^−9^, respectively). Controlling for lineage, fraction of LOH was different in the treatment-refractory and benign corticotroph PitNETs (two-sided Wilcoxon rank sum, *p* = 7.3 × 10^−4^) and there was a trend toward a difference in fraction of LOH in the treatment-refractory and benign lactotroph tumors (two-sided Wilcoxon rank sum, *p* = 0.10), Fig. 2b. Finally, a higher fraction of LOH was found in tumors with *TP53* mutations (one-sided Wilcoxon rank sum, *p* = 3.3 × 10^−8^), consistent with genomic instability from dysregulation of the TP53 pathway [5, 47].

We observed recurrent LOH at the chromosomal level in the treatment-refractory tumor, where LOH of an individual chromosome is defined as LOH involving more than 75% of the chromosome length. While the lactotroph PitNETs displayed higher fraction of LOH without a specific pattern, corticotroph PitNETs had a striking pattern of LOH involving chromosomes 1, 2, 3, 4, 6, 10, 11, 15, 17, 18, 21, and 22. This specific pattern of recurrent chromosomal LOH (rcLOH), was observed in 78% (11/14) of the aggressive, treatment-refractory corticotroph PitNETs compared to only a single (1/14) benign corticotroph tumor (one-sided Fisher’s exact test, *p* = 1.7 × 10^−4^), Fig. 2a. Interestingly, in 11 of the 12 corticotroph tumors with rcLOH, the loss of at least 9 chromosomes occurred in the same proportion of cells (defined as within 10% of the cellular fraction) in a given sample, consistent with either a single event or the LOH of each chromosome being driven to clonality over time (example in Supplementary Fig. 2a). Notably, the Crooke Cell (B-55) and silent ACTH (B-56 and B-57) PitNETs in the benign subset did not demonstrate rcLOH, Fig. 2a. In contrast to benign PitNETs, the majority of aggressive, treatment-refractory PitNETs are Loss of Heterozygosity-High (LOH-H), defined as exhibiting a fraction of LOH above the median for the cohort (fraction of LOH above 0.03), with 48% (11/23) displaying rcLOH, Fig. 2c.

**Fig. 3:**
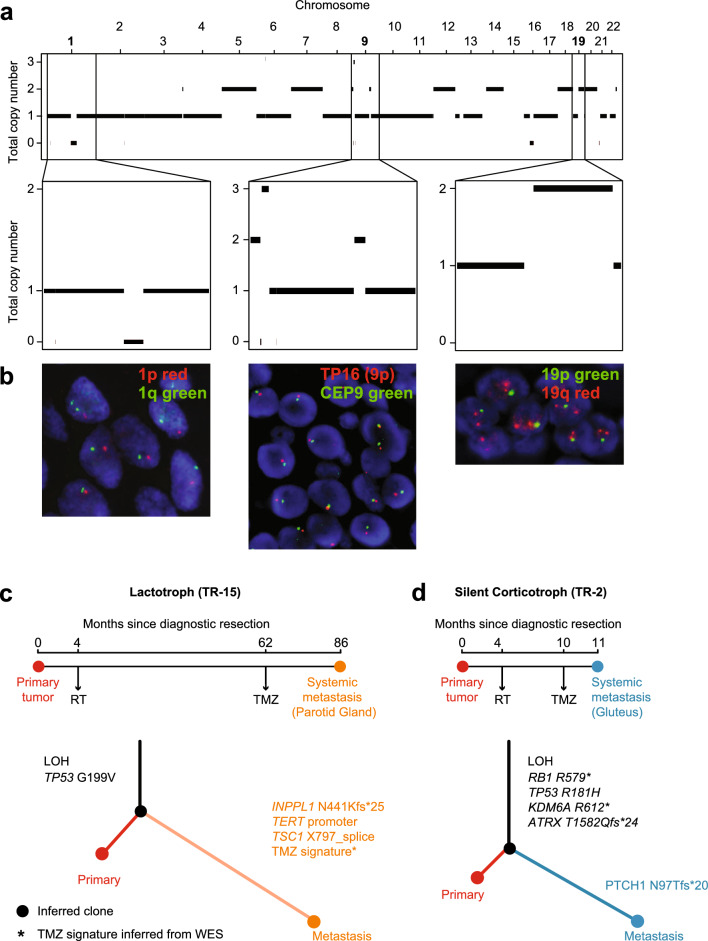
Hypodiploidy is identified by sequencing and fluorescence in situ hybridization. (**a**) Integer total copy number of patient TR-9 estimated by FACETS, displaying extensive LOH including recurrently lost chromosomes across the treatment-refractory cohort. Chromosomes 1, 9, and 19 highlighted to compare to FISH. (**b**) Fluorescence in situ hybridization (FISH) analysis on patient TR-9. Probes against 1p, 1q, 19p, 19q, and CEP9 demonstrate that loss of one copy of chromosome 1, 9, and a segment of 19 including 19p validating the FACETS integer copy-number estimates. (**c**) and (**d**) provides schematic timelines, which outline the clinical course, in a patient with a treatment-refractory lactotroph (TR-15) and a treatment-refractory corticotroph (TR-2) PitNET, respectively. Clone trees from the two patients highlight mutations, LOH and signatures that are either shared or unique to the primary tumor and a metastasis

To confirm whether these LOH events are due to heterozygous loss of the relevant chromosomal regions versus copy neutral LOH, we performed fluorescent in situ hybridization (FISH) in five patients using various probes, including probes that target chromosomes identified as LOH via FACETS, to confirm the copy-number events in each sample, as well as the clonality of these events. Whole-exome sequencing data from patient TR-9 indicated rcLOH, including LOH of chromosomes 1 and 9 and LOH of 19p but not 19q, Fig. 3a. FISH of the chromosome arms of chromosomes 1, 9, and 19 recapitulated the estimated integer copy-number estimates from the genomics by demonstrating the loss of one copy of chromosomes 1, 9 and 19p, Fig. 3b. These findings provide evidence that the chromosomal LOH identified by FACETS are due to loss of the relevant chromosomes or chromosomal regions, which is indicative of an aneuploid genomic profile with multiple monosomies, i.e., hypodiploidy. In all five tumors on which we performed FISH, heterozygous regions hybridized with two probes, whereas chromosomes with LOH hybridized to a single probe, Fig. 3 and Supplementary Fig. 3a–d. In two of the patients, a minor population (less than 15%) of tumor cells showed a duplicated FISH signal pattern; the use of centromere probes supports that these duplicated patterns represent additional chromosomal copies and possibly clonal evolution, Supplementary Fig. 3b, c. Six of 14 patients with treatment-refractory corticotroph and three of seven treatment-refractory lactotroph tumors that were LOH-H were sequenced prior to radiation treatment, establishing that this is not a radiation therapy-driven genomic aberration, Fig. 2a. Sequential resections from eight patients, five of which had resections before and after radiation treatment, further demonstrated that LOH is stable despite treatment, Supplementary Fig. 2b. Patients TR-15 and TR-2 (Fig. 3c, d) have lactotroph and corticotroph PitNETs, respectively, each with LOH involving multiple chromosomes and co-occurring oncogenic alterations thought to represent early events. In both patients, LOH was observed in the radiation therapy-naïve tumor samples along with *TP53* mutation, suggesting that the chromosomal LOH is an early event in the development of pituitary tumors with aggressive clinical behavior.

### Random forest modeling genomic markers of future treatment-refractory behavior

To further explore which clinical and genomic characteristics predict aggressive, treatment-refractory behavior in PitNETs, we implemented a machine learning approach using a random forest algorithm (RF). In this analysis, the prospective and retrospective groups were pooled and randomly split into training and test sets. The training dataset consisted of 17 treatment-refractory and 47 benign tumors, while the test dataset consisted of 6 treatment-refractory and 20 benign tumors. The RF algorithm classifies tumors into treatment-refractory or benign based on an ensemble of decision trees [10]. The variance of 27 features, both genomic and clinical, was examined in the training dataset. Out of 27, only 22 features had non-zero variance (fraction CNA, fraction LOH, sex, lineage, *TP53* status, and LOH status for 17 of the chromosomes) and were used in the RF model. The model performance reported an out-of-bag (OOB) error of 14.06%.

**Fig. 4:**
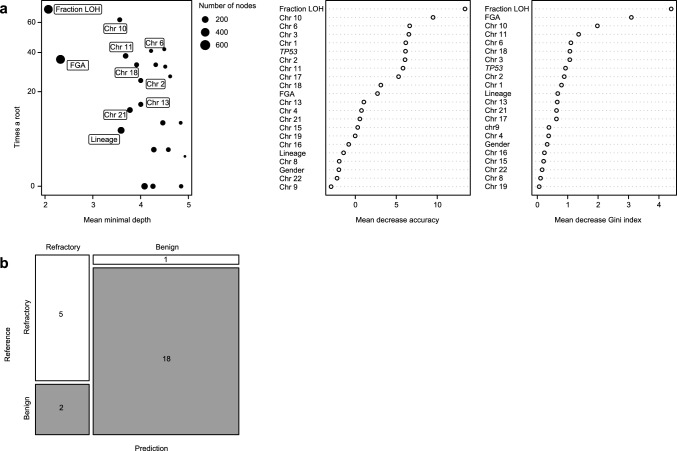
Performance of random forest classifier. (**a**) On the left, the number of times a feature is the root of a regression tree is plotted against the average depth of the first node for that feature (mean minimal depth) with the size of each data point representing total number of nodes that utilize the feature for splitting; the fraction of LOH is the top feature for partitioning aggressive, treatment-refractory behavior. Fraction of LOH is also the top feature for predicting aggressive, treatment-refractory behavior when ranking each feature by the mean decrease in accuracy (middle) and Gini index (right). (**b**) Plot showing the accuracy and precision of our model on the test dataset with prediction on the *x*-axis and the reference truth on the *y*-axis

RF models can be used to identify the most important feature for classification. When applied to the training set, the RF model showed that fraction of LOH was most frequently chosen as a root by the RF classifier among all the features tested (*n* = 22). It outperformed other features by several metrics, including mean minimal depth, the mean decrease in accuracy, and the mean decrease in Gini index, Fig. 4a. Cut point analysis performed on the training set with 500 bootstraps identified fraction of LOH value of 0.11 as the optimal threshold value when optimizing for the highest sum of sensitivity and specificity. At this threshold, the sensitivity was 0.82 and the specificity was 0.91, respectively, as estimated on the training set, Supplementary Fig. 4a. We then compared the performance of three models on the test set (*n* = 26): the RF model, a binary classifier based on fraction of LOH value of 0.11, and a binary classifier of *TP53* mutational status, which was the most common gene-level event observed in the aggressive, treatment-refractory PitNETs. ROC analysis comparing the three classifiers showed that the binary classifier of the fraction of LOH greater than 0.11 segregated treatment-refractory from benign tumors slightly more successfully (AUC = 0.87) than the random forest model (AUC = 0.86), and much better than *TP53* mutational status alone (AUC = 0.75), Supplementary Fig. 4b–d.

When a fraction of LOH-based classifier is applied to the test set, the accuracy of predictions reached 0.88 (95% CI: 0.70–0.96), with sensitivity and specificity values of 0.83 and 0.90, respectively. Positive prediction value was 0.71 while negative prediction value was 0.95. The test data set included six aggressive, treatment-refractory tumors, of which five (83%) were correctly classified as treatment-refractory based on the model. Only one case was misclassified as benign; this misclassified tumor is a somatotroph with a *GNAS* R201S mutation that has grown indolently following radiotherapy. Out of 20 benign cases, 18 (90%) were correctly classified and two cases were predicted to be treatment-refractory, Fig. 4b. The two cases that were benign at the time of last follow-up but classified as treatment-refractory were a gonadotroph (fraction of LOH = 0.31) and a lactotroph tumor (male, fraction of LOH = 0.46).

## Discussion

A rare subset of PitNETs progress following treatment with surgery, conventional medical therapies, and radiation, and can be considered both aggressive and treatment-refractory. At present, there are limited predictive biomarkers of future aggressive, treatment-refractory behavior.

Our data show that aggressive, treatment-refractory PitNETs have an increased fraction of LOH, which is often due to chromosomal losses. In the treatment-refractory corticotroph tumors in our sequencing cohort, LOH of at least 9 chromosomes occurred in the same proportion of cells, raising the possibility that these monosomies occurred as a single event. This recurrent chromosomal LOH (rcLOH) has been reported by other authors but has not been associated with treatment-refractory behavior. Uzilov et al. [50] described 3 corticotroph PitNETs and Bi et al. [9] described a solitary tumor with hypodiploid genomes consistent with rcLOH. Data on the clinical course of tumors are lacking, aside from one patient with rcLOH in the Uzilov paper who had the highest Ki-67 index (23.5%) and the greatest number of recurrences in the cohort. In a paper by Lasolle et al., only a very small subset of corticotroph PitNETs had a pattern of deletions on array comparative genomic hybridization that is potentially consistent with rcLOH [26]. This study was unable to find an association between copy-number variation (CNV) and tumor recurrence. An association between CNV and treatment-refractory behavior was not investigated for two possible reasons: (1) insufficient follow-up and (2) the rarity of this clinical phenotype. Finally, in the paper by Neou et al., 86 PitNETs were analyzed using a single-nucleotide polymorphism (SNP) array. This analysis included 13 pituitary tumors that were considered aggressive and did not identify an association between chromosomal alterations and aggressiveness; however, it is unknown whether these “aggressive” tumors progressed following treatment with an initial course of radiotherapy and fulfill the definition of treatment-refractory used in our study [38].

While an association between fraction of genome altered (FGA) and aggressive, treatment-refractory behavior was identified in our cohort, the random forest (RF) model shows that fraction of LOH outperforms FGA. In this study, we identify a genomic marker of aggressiveness that has not been identified by others because of differences in the endpoints that were investigated both genomic (FGA vs. LOH) and clinical (recurrence vs aggressiveness vs treatment-refractoriness), and because our cohort includes a greater number of tumors with high malignant potential. We believe that the high number of tumors with rcLOH in our cohort compared to others is due to this enrichment of very aggressive tumors.

The stability and the early timing of this loss of heterozygosity are supported by the consistency of the losses across multiple resections from the same patient, including the diagnostic tumor samples. We show that LOH in PitNETs associates with *TP53* mutations, which is an expected finding, given that *TP53* mutations have been associated with aneuploidy across cancer types [47].

Aneuploidy occurs in up to 90% of solid tumors and is known to vary by tumor type [7]. It has been shown that tumors arising from the same tissue type and tumors arising from similar tissue types share similar patterns of aneuploidy [7, 21]; for example, squamous cancers of multiple primary sites cluster together [21, 22]. Additionally, tissues adjacent to tumors and even healthy tissues have been found to harbor copy-number alterations that are similar to the alterations observed in tumors from corresponding tissues [3, 17]. The rcLOH phenotype that was identified in the treatment-refractory corticotroph PitNETs shares some similarity to the pattern of LOH identified in normal pituitary tissue, including loss of chromosomes 1, 2, 11, 18, 21, and 22 [17]. However, this rcLOH phenotype identified in corticotroph PitNETs more closely resembles a pattern of LOH found in pancreatic neuroendocrine tumors with a more aggressive phenotype (characterized by loss of chromosomes 1, 2, 3, 6, 8, 10, 11, 16, 21, and 22) [43] and other endocrine neoplasms, such as adrenocortical carcinoma [54]. This suggests that rcLOH in PitNETs is more indicative of an endocrine tumor type, rather than pertaining specifically to this tumor’s adenohypophyseal tissue of origin. In the context of an endocrine cell type, a hypodiploid genome due to multiple monosomies on specific chromosomes may represent a critical driver due to haploinsufficiency and disruption of signaling pathways [27].

rcLOH occurred in the vast majority of treatment-refractory corticotroph PitNETs and only in a single patient with a benign corticotroph tumor. This patient (B-54) with the benign corticotroph tumor with rcLOH underwent surgical resection and the pathology was unremarkable, with a Ki-67 of 1%. Besides rcLOH and a *TP53* mutation, this tumor was genomically quiescent without additional oncogenic drivers and has not recurred thus far, after 7 years of follow-up, possibly because the tumor was confined to the gland permitting a complete resection. Patient TR-9 (Supplementary Figs. 1a and 2b) with a treatment-refractory metastatic PitNET started as a similar tumor with rcLOH without additional oncogenic drivers, with a Ki-67 of  ~ 1%. Unlike patient B-54, patient TR-9 could only undergo a subtotal resection due to the tumor’s invasive behavior. The recurrent tumor acquired *CCND3* amplification on a subsequent resection, followed by the acquisition of *CDKN2A/B* loss, mismatch repair deficiency, and alkylator-induced somatic hypermutation, which was identified in a liver metastasis that had a Ki67 of 50% [32].

Aggressive, treatment-refractory corticotroph PitNETs also had mutations in *ATRX* and *DAXX*, which are responsible for alternative lengthening of telomeres (ALT). This finding is consistent with prior studies [12, 20], which identified ALT in recurrent PitNETs, and explains the absence of tumors with *ATRX* and *DAXX* mutations in the benign tumors in our prospective cohort. Additionally, the cohort includes tumors with mutations in MMR genes in the diagnostic resection; the functional significance of these mutations is supported by the loss of protein expression on MMR IHC, high mutational burden, and the MSI-high/MSI-intermediate status of these tumors. This finding is of significant interest, given that the PD1 inhibitor pembrolizumab is approved for the treatment of mismatch repair deficient tumors, agnostic to tumor type. Notably, dramatic responses to checkpoint inhibitors have been reported in the setting of mismatch repair deficiency in PitNETs [32, 44].

Among lactotrophs, we observed a trend toward higher LOH in the treatment-refractory subset. A high fraction of LOH is more consistently seen than hotspot mutations in *SF3B1*, which has been reported in recurrent lactotrophs [28], but was only identified in one treatment-refractory lactotroph in our cohort. The only recurrent gene level event that we identified in treatment-refractory lactotroph tumors were mutations in *TP53.*

Compared to prior sequencing efforts, this study captures patients with more aggressive PitNETs based on well-documented clinical behavior. Given the rarity of treatment-refractory behavior, this study uncovers genomic events that have not been well described in PitNETs, including a phenotype that has been observed in other endocrine tumors. A machine learning approach using random forest (RF) shows that future aggressive, treatment-refractory behavior can be predicted and that fraction of LOH is the best individual predictive marker of future treatment-refractoriness. This work also demonstrates that treatment-refractory PitNETs are genomically distinct from an unselected group of surgical patients. However, given the limited number of tumors included, this study may not have captured the full range of behavior found in patients with PitNETs. A larger, prospective cohort with longer periods of clinical follow-up is therefore needed to validate these findings.

LOH is a common feature of aggressive, treatment-refractory PitNETs and appears to have prognostic significance. The importance of this phenotype may extend beyond prognostication as LOH, and rcLOH in particular, seems to be a fundamental molecular feature, which may have functional implications that can be leveraged in the treatment of this uncommon malignancy.

### Supplementary Information

Below is the link to the electronic supplementary material.

Supplementary file1 (PDF 9358 KB)

## Data Availability

The whole-exome sequencing will be deposited in NCBI dbGaP (Accession #phs001783: Exome Recapture and Sequencing of Prospectively Characterized Clinical Specimens from Cancer Patients; https://www.ncbi.nlm.nih.gov/projects/gap/cgi-bin/study.cgi?study_id=phs001783.v1.p1). The somatic mutational and clinical data from targeted next-generation sequencing have been deposited for visualization and analysis in the cBioPortal for Cancer Genomics (https://www.cbioportal.org/).
