# Supplementary material for: Genome-wide loss of heterozygosity predicts aggressive, treatment-refractory behavior in pituitary neuroendocrine tumors
Source: Acta Neuropathol. 2024 May 17;147(1):85. doi: 10.1007/s00401-024-02736-8 (PMC11101347; doi:10.1007/s00401-024-02736-8)

Title: Genome-wide loss of heterozygosity predicts treatment-refractory behavior in pituitary neuroendocrine tumors

Journal: *Acta Neuropathologica*

Authors: Andrew L. Lin[1-3,*], Vasilisa A. Rudneva[4,*], Allison L. Richards[4,*], Yanming Zhang[5], Hyung Jun Woo[4], Marc Cohen[1,3,6], Jamie Tisnado[7], Nazanin Majd[8], Sharon L. Wardlaw[9], Gabrielle Page-Wilson[9], Soma Sengupta[10], Frances Chow[11], Bernard Goichot[12], Byram H. Ozer[13], Jorg Dietrich[14], Lisa Nachtigall[15], Arati Desai[16], Tina Alano[4], Shahiba Ogilive[1], David B. Solit[4,17], Tejus A. Bale[5], Marc Rosenblum[5], Mark T.A. Donoghue[4,**, †], Eliza B. Geer[1,3,17,**, †], Viviane Tabar[1,3**]

[1] Department of Neurosurgery, Memorial Sloan Kettering Cancer Center, New York, NY
[2] Department of Neurology, Memorial Sloan Kettering Cancer Center, New York, NY
[3] Multidisciplinary Pituitary and Skull Base Tumor Center, Memorial Sloan Kettering Cancer Center, New York, NY
[4] Marie-Josée and Henry R. Kravis Center for Molecular Oncology, Memorial Sloan Kettering Cancer Center, New York, NY
[5] Department of Pathology and Laboratory Medicine, Memorial Sloan Kettering Cancer Center, New York, NY
[6] Department of Surgery, Memorial Sloan Kettering, Cancer Center, New York, NY
[7] Department of Radiology, Memorial Sloan Kettering, Cancer Center, New York, NY
[8] Department of Neuro-oncology, The University of Texas MD Anderson Cancer Center, Houston, TX
[9] Department of Medicine, Columbia University Irving Medical Center, New York, NY
[10] Department of Neurology and Neurosurgery, University of North Carolina, Chapel Hill, NC
[11] Department of Neurology, Keck School of Medicine at University of Southern California Medical Center, Los Angeles, CA
[12] Department of Endocrinology, Les Hôpitaux Universitaires de Strasbourg, Strasbourg, FR
[13] Department of Oncology, Sibley Memorial Hospital/Johns Hopkins, Washington, DC.
[14] Department of Neurology, Massachusetts General Hospital, Boston, MA
[15] Department of Medicine, Massachusetts General Hospital, Boston, MA
[16] Department of Medicine, University of Pennsylvania Medical Center, Philadelphia, PA
[17] Department of Medicine, Memorial Sloan Kettering Cancer Center, New York, NY

†Corresponding authors: Eliza B. Geer (geere@mskcc.org) and Mark T.A. Donoghue (donoghum@mskcc.org), Memorial Sloan Kettering Cancer Center, David H. Koch Center for Cancer Care, 530 East 74th Street, 22nd Floor, Room 22-248, New York, NY 10021. Telephone: (646) 608-3797. Fax number: 646-888-2738.

*Co-first; **Co-senior

Supplementary Fig. 1. Genomic alterations in individual tumor samples. (a) Oncoprint of recurrently altered driver genes in treatment-refractory PitNET patients with multiple sequenced resections in chronological order by time of resection (left to right) on a per patient basis. Patient demographics and clinicopathologic features are on the top, followed by common genetic alterations. Tumors are reported as metastatic or non-metastatic based on the tumor's status at the time of the tumor resection. (b) Gene model of *USP8* mutations identified in the cohort. Only one tumor had a canonical *USP8* gain of function mutation in the 14-3-3 binding domain (nucleotides corresponding to amino acids 715-720).

Supplementary Figure 1

a

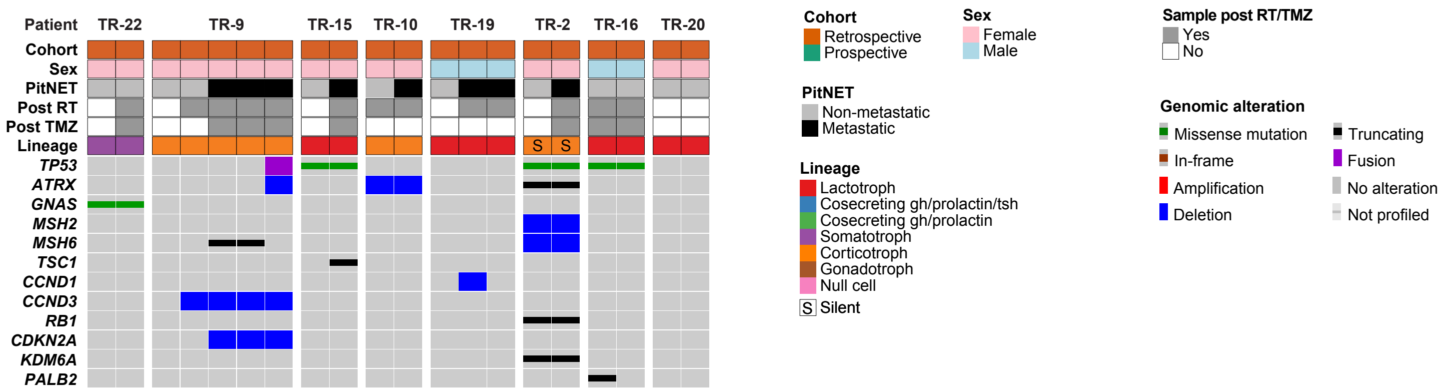

b

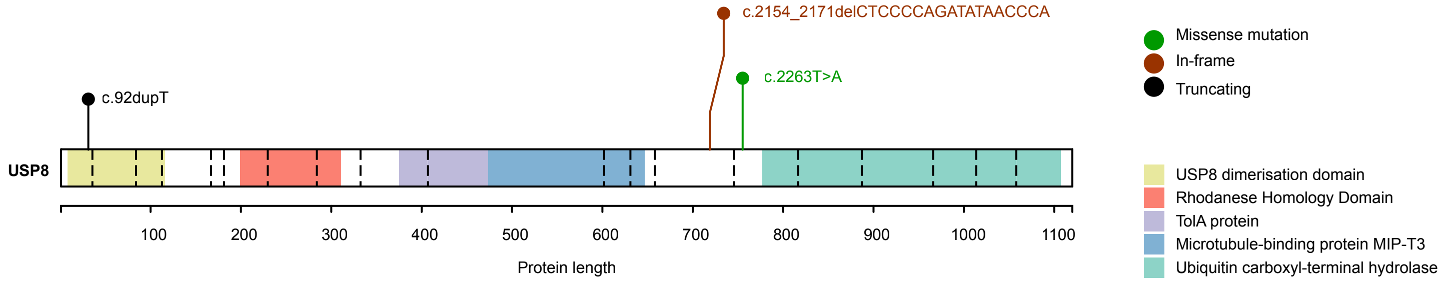

Supplementary Fig. 2. Chromosomal loss of heterozygosity (LOH) as determined by FACETS shows stability across multiple sequenced resections. (a) Integer (total, minor) copy number calls from FACETS analysis of sequencing data from patient TR-10 revealing whole-chromosomal losses of heterozygosity on multiple chromosomes (1, 2, 3, 6, 8, 10, 11, 15, 16, 17, 18, 21, and 22). The estimated cellular fraction profile is at the bottom. The overall tumor ploidy is estimated to be 1.37. The tumor sample purity is estimated to be 0.76. (b) The heatmap shows LOH status for individual chromosomes with each blue box demonstrating LOH covering at least 75% of the given chromosome in treatment-refractory PitNET patients with multiple sequenced resections in chronological order by time of resection (left to right) on a per patient basis. Patient demographics and clinicopathologic features are on the top. Tumors are reported as metastatic or non-metastatic based on the tumor's status at the time of the tumor resection.

Supplementary Figure 2

a

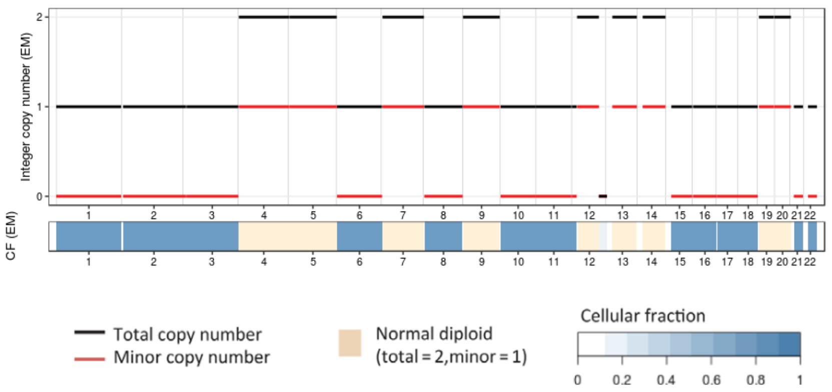

b

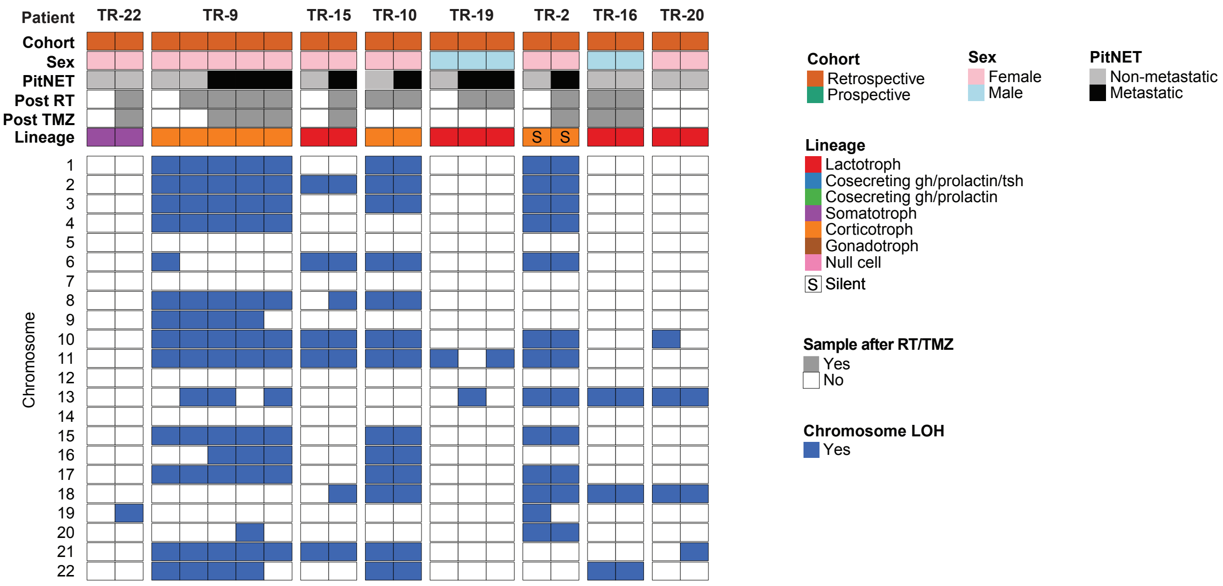

Supplementary Fig. 3. Hypodiploidy is identified by sequencing and fluorescence in situ *hybridization.* (a) Integer total copy number of patient TR-1 estimated by FACETS. Chromosomes 1, 7,12, and 15 are highlighted to compare against FISH, which was performed using probes against 1p, 1q, CEP7, MET, MDM2, CEP12, and NTRK3 (break-apart probe with a fusion signal). (b) Integer total copy number of patient TR-4 estimated by FACETS. Chromosomes 1, 7, and 22 are highlighted to compare against FISH, which was performed using probes against 1p, 1q, CEP7, MET, and EWSR1 (break-apart probe with a fusion signal). In the experiment performed using MET/CEP7 probes, a small population of tumor cells have a duplicated signal pattern. (c) Integer total copy number of patient TR-15 estimated by FACETS. Chromosomes 1, 2, and 19 are highlighted to compare against FISH, which was performed using probes against 1p, 1q, MYCN, CEP2, 19p, and 19q; a minority of tumor cells have a duplicated signal pattern. (d) Integer total copy number of patient B-54 estimated by FACETS. Chromosomes 1, 7,12, and 16 are highlighted to compare against FISH, which was performed using probes against 1p, 1q, CEP7, MET, MDM2, CEP12, and FUS (break-apart probe with a fusion signal).

Supplementary Figure 3

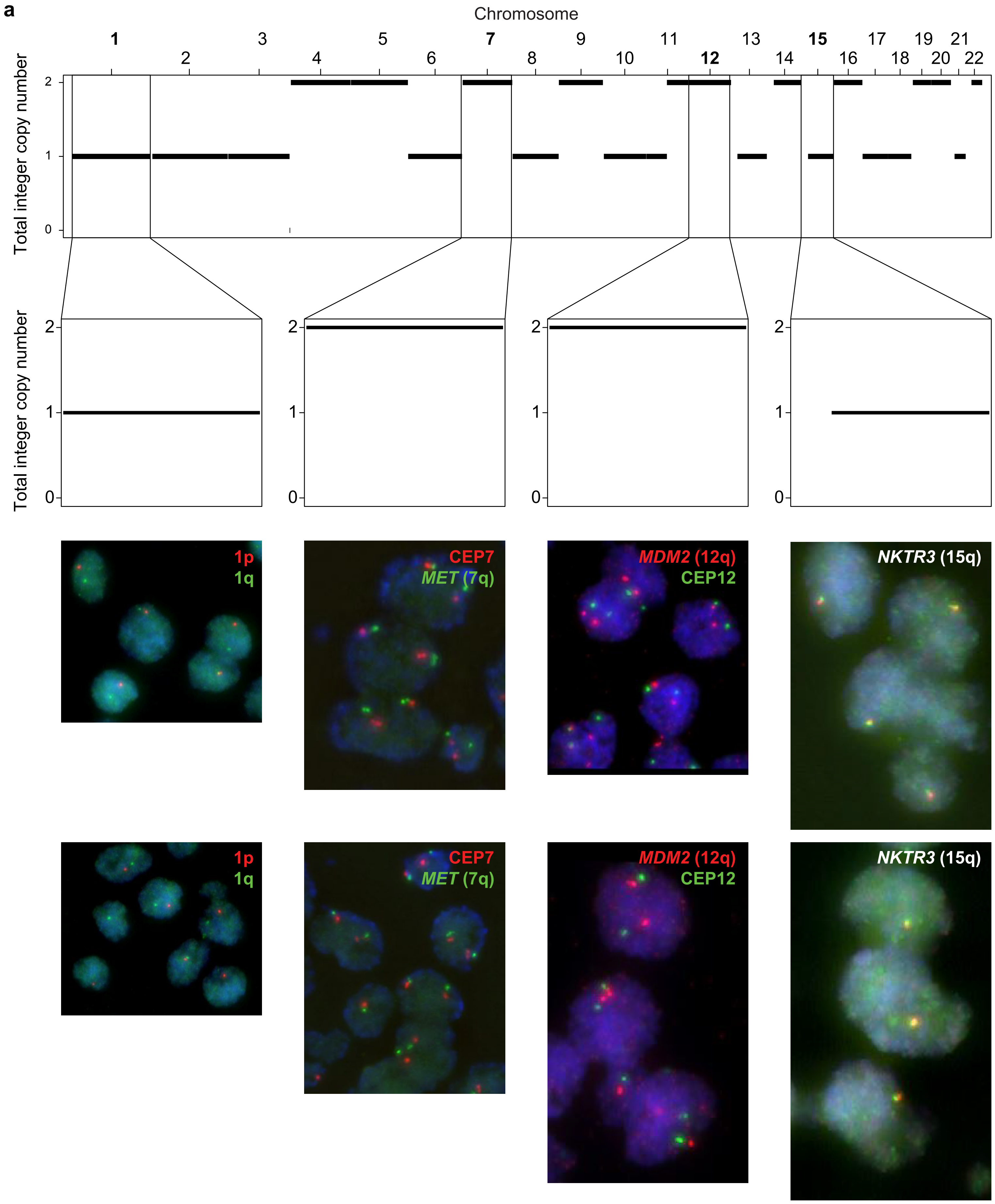

b **Supplementary Figure 3**

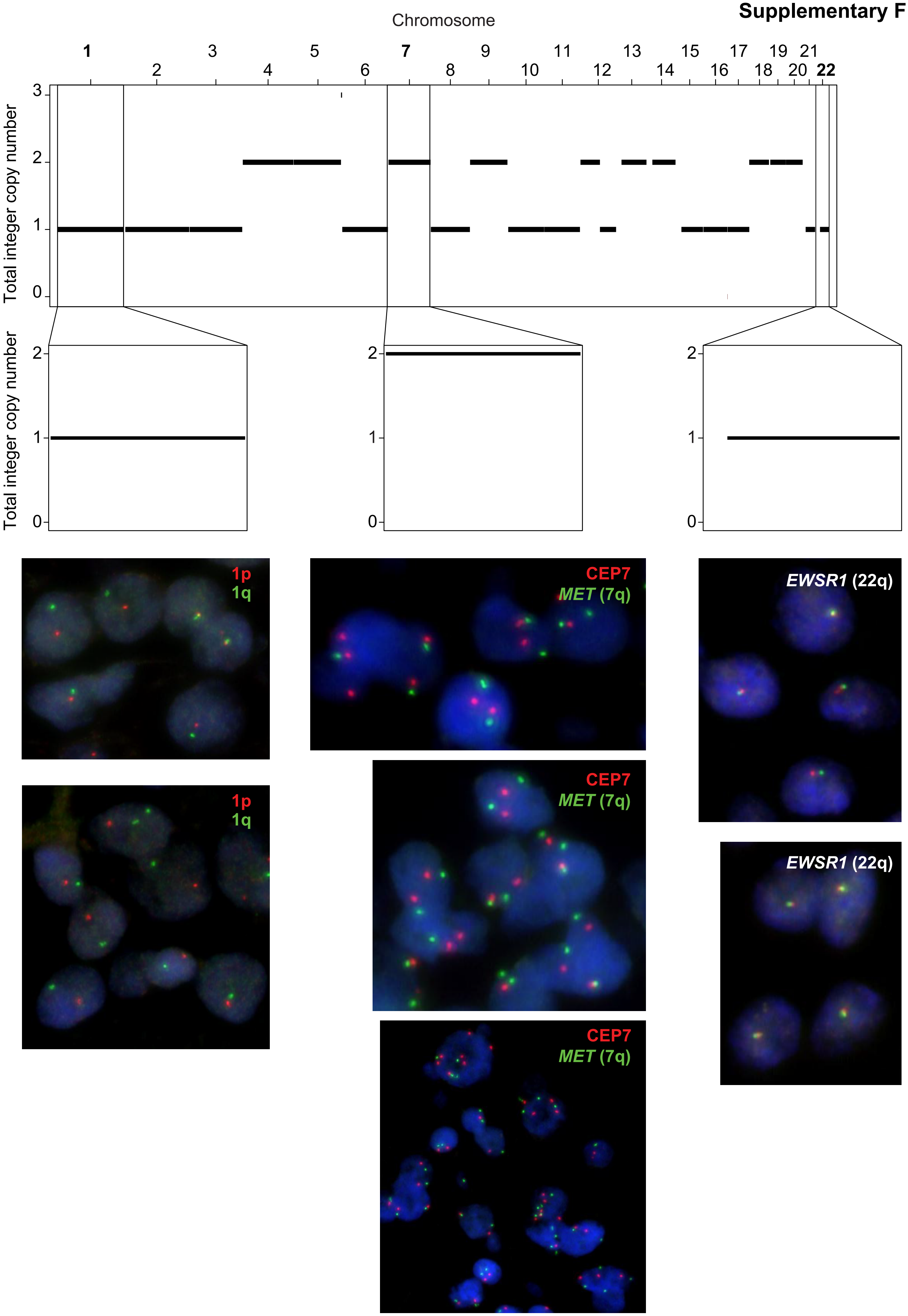

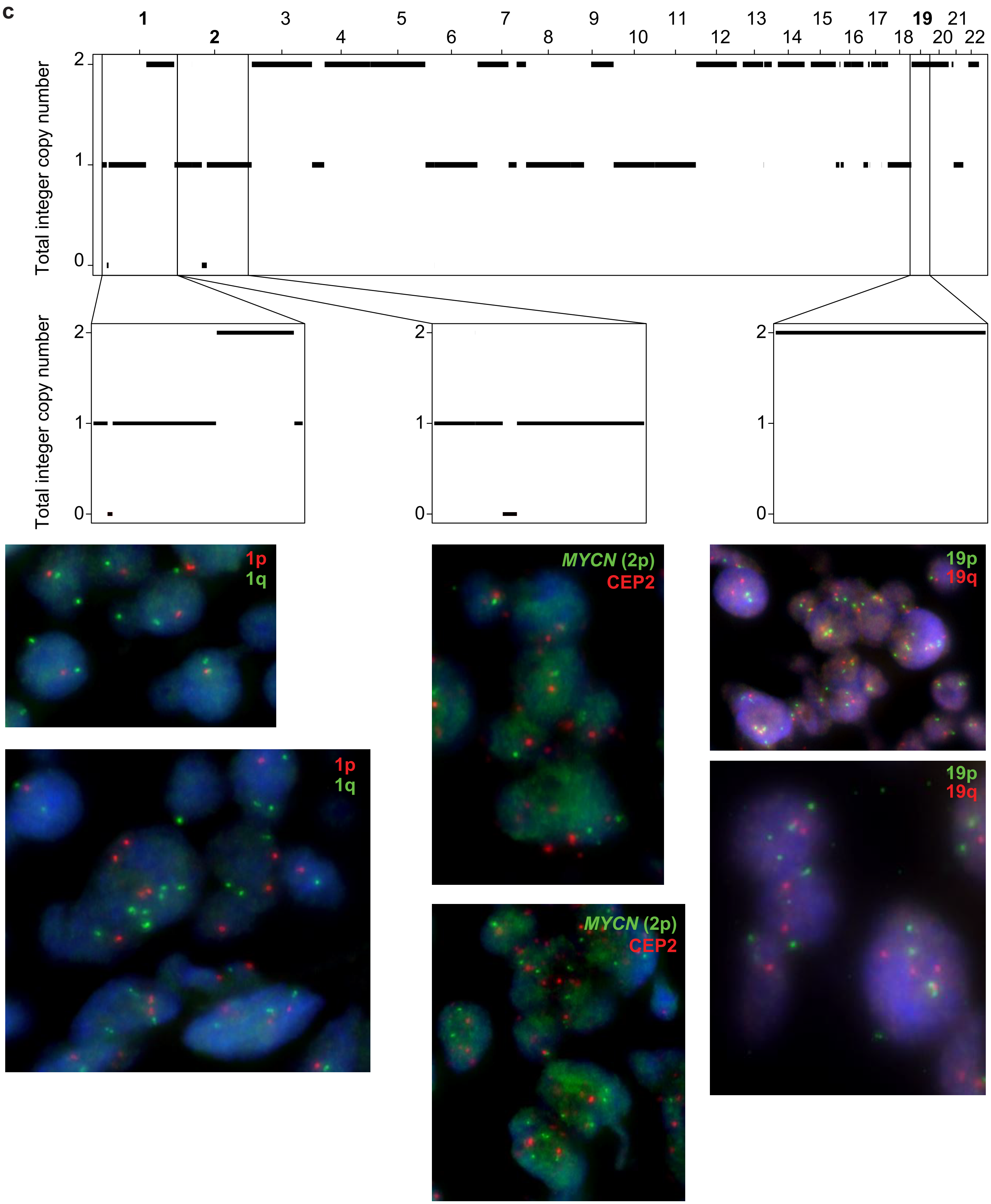
c
1
2
3
4
5
6
7
8
9
10
11
12
13
14
15
16
17
18
19
20
21
22
Total integer copy number
2
1
0
1p
1q
MYCN (2p)
CEP2
19p
19q

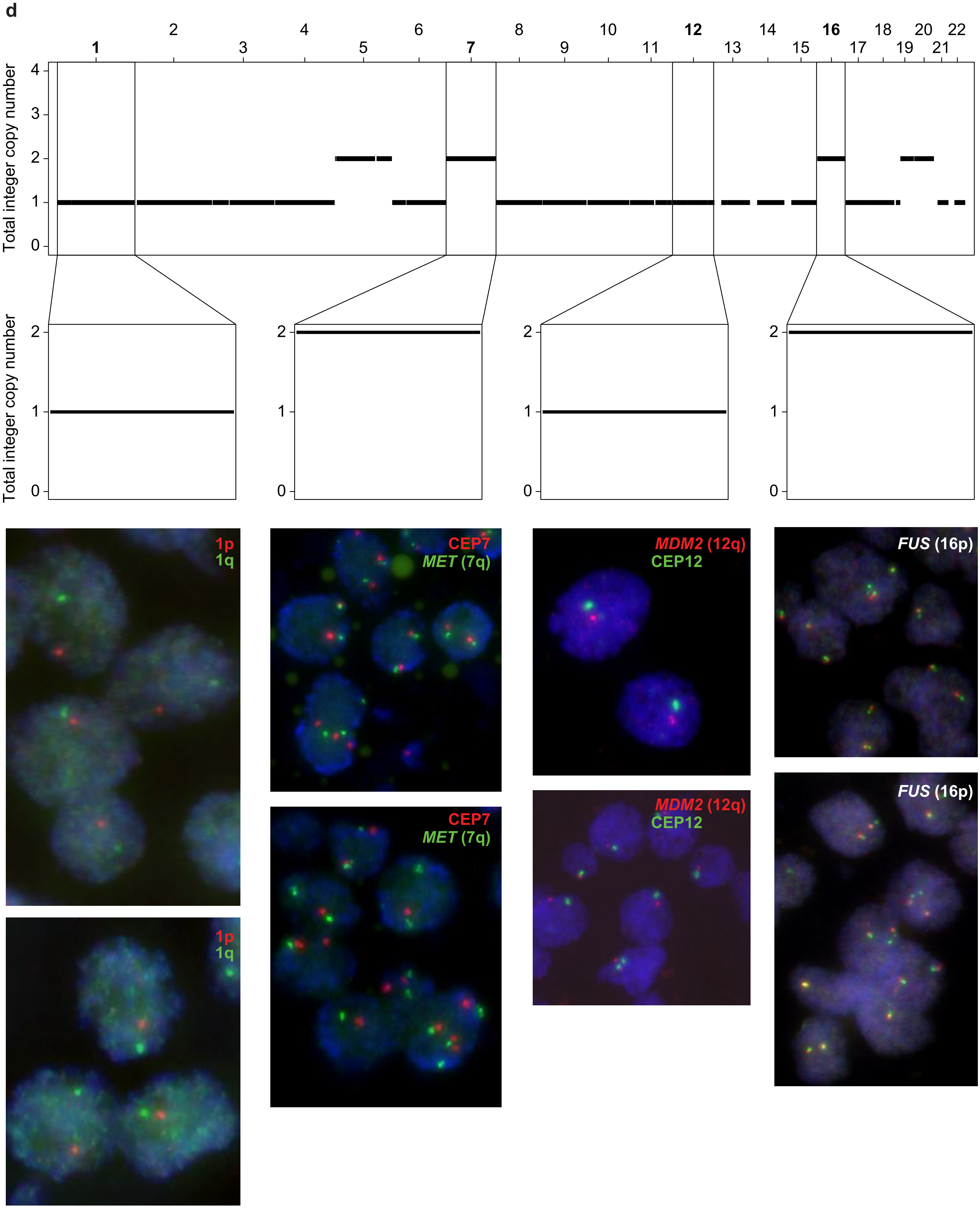
d
1
2
3
4
5
6
7
8
9
10
11
12
13
14
15
16
17
18
19
20
21
22
Total integer copy number
4
3
2
1
0
Total integer copy number
2
1
0
1p
1q
CEP7
MET (7q)
MDM2 (12q)
CEP12
FUS (16p)

Supplementary Fig. 4. Receiver operating characteristic (ROC) analysis comparing classifiers. (a) Cut point analysis performed on the training set with 500 bootstraps identified fraction of LOH value of 0.11 as the optimal threshold value when optimizing for the highest sum of sensitivity and specificity. The corresponding specificity and sensitivity values calculated for the training set are shown. ROC curves showing the performance on the test set of the (b) random forest model, (c) a binary classifier based on *TP53* mutational status, (d) and a binary classifier based on fraction of LOH value that is above or below 0.11 (cut point identified as optimal). Local maxima and area under the ROC curve (AUC) values are shown for each plot.

Supplementary Figure 4

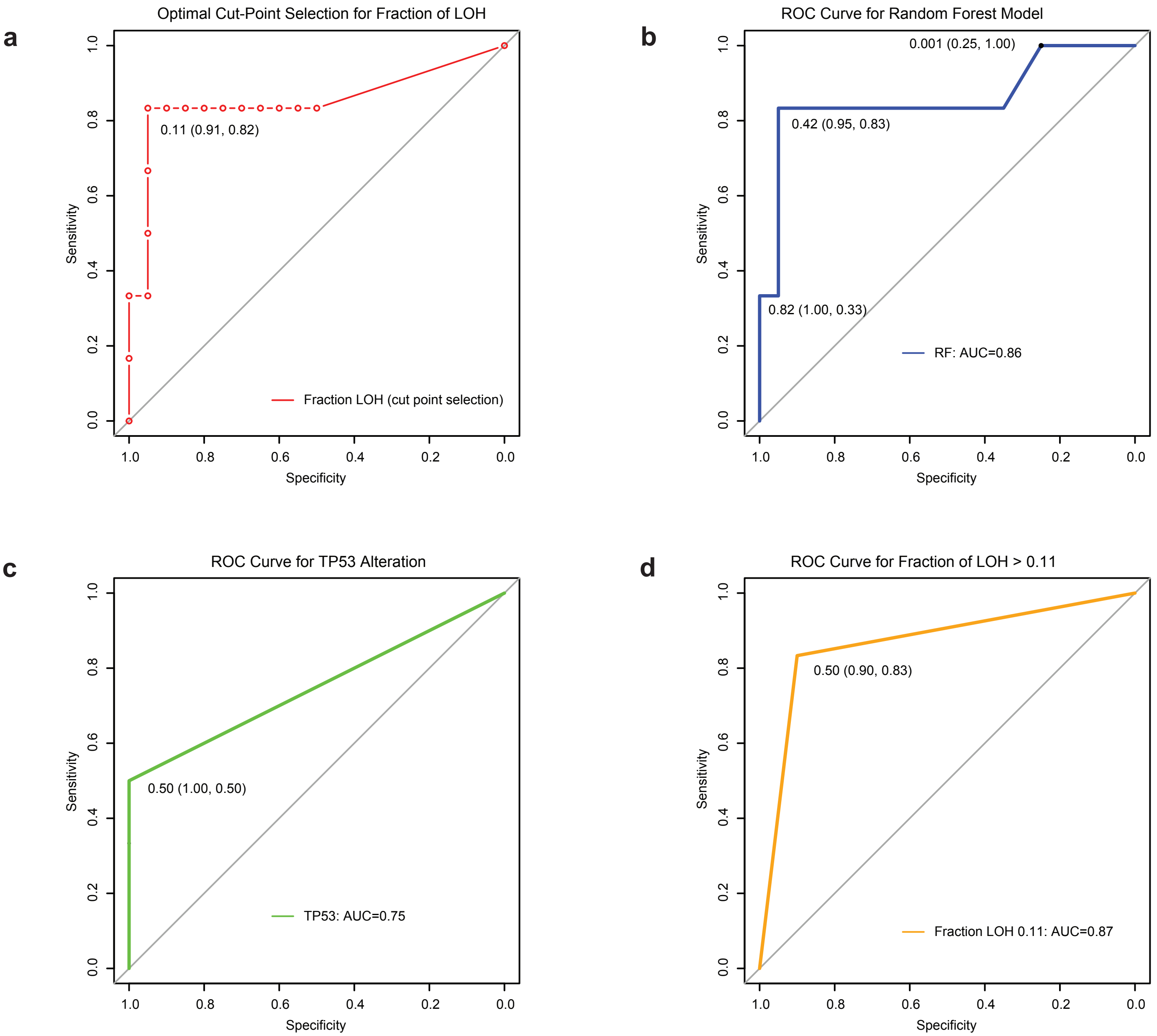

Supplement: Supplementary file 1 — Supplementary file1 (PDF 9358 KB) [file 401_2024_2736_MOESM1_ESM.pdf]
